# *BRCA1/2* Mutations Appear Embryo-Lethal Unless Rescued by Low (CGG n<26) *FMR1* Sub-Genotypes: Explanation for the “*BRCA* Paradox”?

**DOI:** 10.1371/journal.pone.0044753

**Published:** 2012-09-12

**Authors:** Andrea Weghofer, Muy-Kheng Tea, David H. Barad, Ann Kim, Christian F. Singer, Klaus Wagner, Norbert Gleicher

**Affiliations:** 1 The Center for Human Reproduction, New York, New York, United States of America; 2 Department of Gynecological Endocrinology and Reproductive Medicine, Medical University Vienna, Vienna, Austria; 3 Department of Obstetrics and Gynecology, Medical University Vienna, Vienna, Austria; 4 Foundation for Reproductive Medicine, New York, New York, United States of America; 5 Department of Human Genetics, Medical University Graz, Graz, Austria; The Chinese University of Hong Kong, Hong Kong

## Abstract

*BRCA1/2* mutations and recently described constitutional *FMR1* genotypes have, independently, been associated with prematurely diminished ovarian reserve. Whether they interrelate in distribution, and whether observed effects of *BRCA1/2* and *FMR1* on ovaries are independent of each other, is unknown. In a prospective comparative cohort study, we, therefore, investigated the distribution of constitutional *FMR1* genotypes, normal (*norm*), heterozygous (*het*) and homozygous (*hom*), and of their respective sub-genotypes (*high*/*low*), in 99 *BRCA1/2* mutation-positive women and 410 female controls to determine whether distribution patterns differed between study and control patients. In contrast to controls, *BRCA1/2* carriers demonstrated almost complete absence of all constitutional *FMR1* genotypes except for sub-genotypes with *low* (CGG _n<26_) alleles. Cross tabulation between *BRCA1/2*-positive patients and controls confirmed significant group membership, related to *FMR1* distribution (P<0.0001). These results offer as most likely explanation the conclusion that *BRCA1/2* mutations are embryo-lethal, unless rescued by *low* (CGG _n<26_) *FMR1* sub-genotypes, present in approximately one quarter of all women. Women with *low FMR1* sub-genotypes, therefore, should reflect increased *BRCA1/2*-associated cancer risks, while the remaining approximately 75 percent should face almost no such risks. If confirmed, this observation offers opportunities for more efficient and less costly *BRCA1/2* cancer screening. The study also suggests that previously reported risk towards prematurely diminished ovarian reserve in association with *BRCA* mutations is *FMR1*-mediated, and offers a possible explanation for the so-called “*BRCA* paradox” by raising the possibility that the widely perceived *BRCA1/2*-associated tumor risk is actually *FMR1*-mediated.

## Introduction

The fragile X mental retardation 1 (*FMR1*) gene, located on the long arm of the X chromosome (Xq27.3) at base pairs 146,801,200 to 145,840,302, contains a repetitive DNA segment, the CGG_n_ trinucleotide. The gene has, historically, primarily been investigated due to associated neuro-psychiatric risks at so-called premutation range CGG expansions (approximately CGG _n = 55–200_) and at full mutation range (CGG _n>200_), the so-called fragile X syndrome [1].

In women, the premutation range genotype of *FMR1* has for decades been known associated with greatly increased risk towards premature ovarian failure (POF), often also called primary ovarian insufficiency (POI) [2]. The gene until recently was, however, not known for any specific associated ovarian phenotypes. This changed with the description of newly described constitutional, so-called ovarian genotypes of *FMR1*, with distinct phenotypical ovarian aging patterns, associated with prematurely diminished functional ovarian reserve and other associations [3], [4].

These newly described ovarian genotypes of *FMR1* were based on definition of a normal CGG_n_ range of 26–34 (median CGG _n = 30_) [3], later confirmed to be identical in all races, though in outliers (*het* and *hom* genotypes and sub-genotypes) demonstrating distinct distribution differences between races [5], [6]. The median of CGG _n = 30_ corresponded with the switching point between positive and negative message and peak translation of the gene product of *FMR1*, as previously reported by Chen et al [7].

These new ovarian genotypes were also shown associated with IVF pregnancy chances, and to define risk towards autoimmunity in infertile patient populations [4], [6].

Observing ovarian responses during in vitro fertilization (IVF), Oktay et al suspected in young *BRCA* mutation carriers with breast cancer a similar impairment in functional ovarian reserve as had been previously observed in association with certain *FMR1* genotypes and sub-genotypes and, indeed, demonstrated such an association with *BRCA1* [8]. Considering potential overlaps in *BRCA1/2* and *FMR1* genotypes and sub-genotypes, observed associations with *BRCA1/2*, however, do not necessarily have to be causal and, at least theoretically, could be related to overlapping *FMR1* genotypes and sub-genotypes.

The commonality of prematurely diminished ovarian reserve, reported independently for *BRCA* mutations and the *FMR1* gene, therefore, led us to investigate to what degree *BRCA1/2* and *FMR1* genotypes and sub-genotypes interrelate in distribution, and whether observed *BRCA* effects on ovarian reserve may be *FMR1*-mediated. As this study will demonstrate, the relationship between *BRCA1/2* and the *FMR1* gene was found to be surprisingly interdependent, raising a number of new biological questions of importance.

## Methods

### Study Design

Coordination of research efforts between Austrian and U.S. centers involved one author (A.W.). *BRCA1/2* and *FMR1* data of Austrian *BRCA1/2*-positive patients were obtained in Austria, and without further analysis anonymized forwarded to New York investigators (D.H.B., A. K., N. G.) for statistical analyses.

### Study Groups

The study involved two distinct patient populations: (i) 99 Austrian female *BRCA1* or *BRCA2* mutation-positive patients. Their *BRCA1/2* testing was performed at the Medical University Vienna, Vienna, Austria, while their *FMR1* assays were performed at the Medical University Graz, Graz, Austria. (ii) 410 female infertility patients, whose anonymized clinical information, including *FMR1* testing results, were stored in the electronic research database of the Center for Human Reproduction in New York, U.S.A. An infertile female population like the one presented here, previously was shown to demonstrate similar CGG count distributions as a general population [5], [6].

An initial statistical analysis of the Austrian data set, because of here reported rather extraordinary findings, raised questions about reproducibility of Austrian and U.S. *FMR1* results. The Austrian laboratory was, therefore, requested to provide random anonymized results of a patient population reflecting the whole CGG spectrum. When the U.S. investigators analyzed these 105 additional controls, *FMR1* genotypes and sub-genotypes did not differ significantly in either median or distribution between 25^th^ and 75^th^ percentiles from infertile U.S. controls, thus confirming reproducibility and compatibility of Austrian and U.S. *FMR1* analyses.

### Laboratory Analyses

*BRCA1/2* and *FMR1* analyses of the Austrian study group were performed in Vienna (*BRCA1/2*) and Graz (*FMR1*), Austria, respectively. Until and inclusive of 2008, *BRCA1/2* analyses were performed by denaturation high performance liquid chromatography, as previously reported by the laboratory [9]. After 2008, DNA sequencing, with use of chain-terminating inhibitors, was utilized [10].

*FMR1* analyses in Austria were performed by Southern blot hybridization and polymerase chain reaction (PCR), as previously reported from this laboratory [11]. New York *FMR1* analyses were performed by commercial assays, as previously reported [3]–[6].

Austrian *FMR1* data were reported for both alleles as CGG _n,_ and in New York converted to the recently reported format of ovarian genotypes and sub-genotypes [3], [4], [6]. In brief, it is based on a normal range of CGG _n = 26–34_, with median of 30 repeats. Women, therefore, can have the following genotypes: normal (*norm*) if both alleles are in normal range; heterozygous (*het*), if one allele is in and one outside of normal range; and homozygous (*hom*) if both alleles are outside normal range. *Het* and *hom* genotypes can then be further subdivided, depending whether abnormal alleles are above (*high*) or below (low) normal range into *het-norm/high* and *het-norm/low* and *hom-high/high*, *hom-high/low* and *hom-low/low* sub-genotypes. Because of the small number of *hom* patients, they are not sub-divided into sub-genotypes in this study.

### Institutional Review Board (IRB) Approvals

The Ethikkommission der Medizinischen Universität Wien, an IRB at the University Vienna, Austria, approved analysis of *BRCA1/2* patients. Written consents were obtained from all participants. The CHR’s IRB (Institutional Review Board, the Center for Human Reproduction) approved data analysis of the U.S. control group. CHR patients, at time of initial consultation, sign an informed consent, which allows for review of medical records for research purposes as long as patient anonymity and confidentiality of the medical record are maintained. These conditions were met, and the study, therefore, qualified for expedited review. Patients, undergoing *FMR1* testing at CHR, in addition, sign a genetic testing-specific consent, and all clinical and research staff at CHR, in concordance with federal HIPAA rules, in writing commit to maintaining confidentiality of medical record and anonymity of patients.

### Statistical Analyses

Proportions of *FMR1* genotypes and sub-genotypes were compared between the two study groups using cross-tabulations and calculations of Chi-square and Cramer’s V statistics. When comparing CGG _n_ as a continuous function between the groups, nonparametric testing was used because CGG _n_ in populations tends to be positively skewed. Mann-Whitney U tests were conducted to evaluate differences between the two groups on median change in CGG _n_ of both alleles.

All statistical calculations were performed utilizing SPSS, version 18 (Chicago, Illinois).

## Results

**Figure 1:**
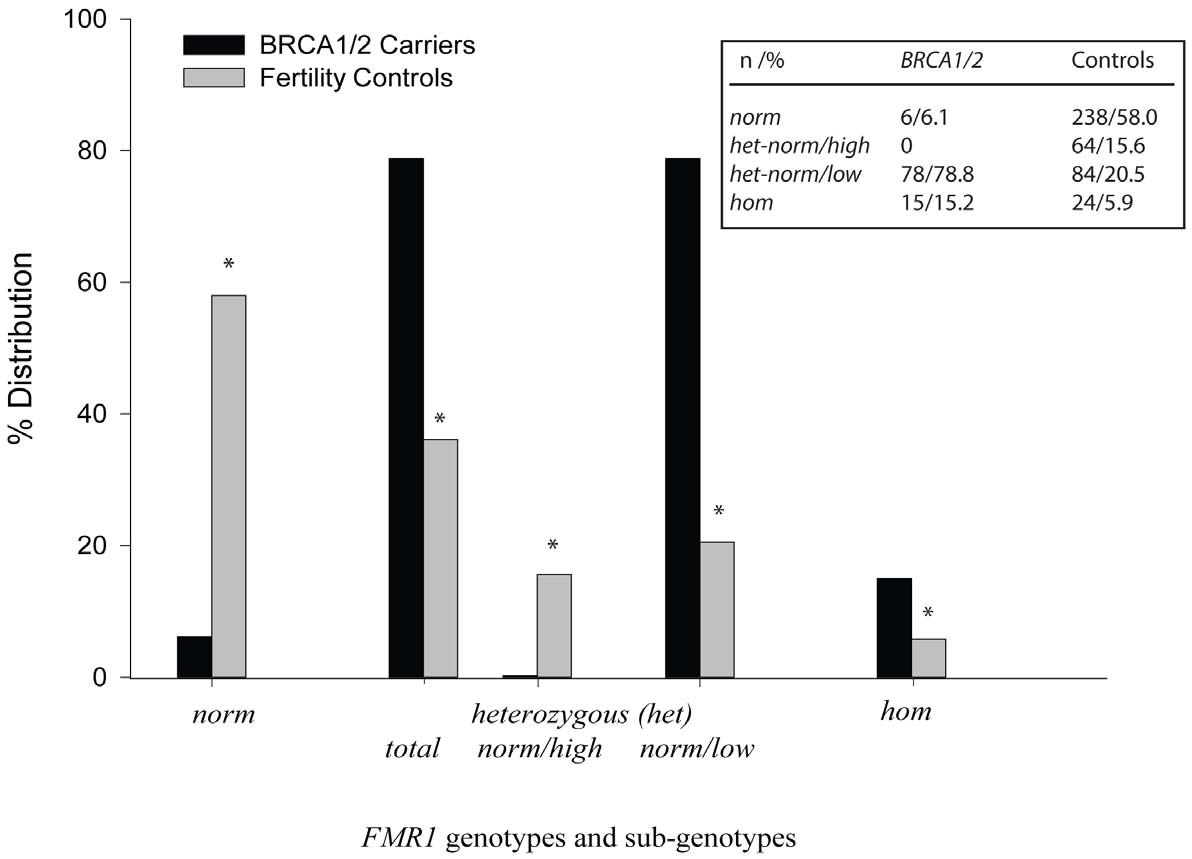
Distribution of *FMR1* genotypes and sub-genotypes in women with *BRCA1/2* mutations (black bars) and U.S. (gray) comparison group; * within each category denotes significance at P<0.05. Noteworthy are the excess of *het-norm/low* and complete absence of *het-norm/high* in *FMR1* sub-genotypes in *BRCA1/2* mutation carriers, and the very low prevalence of women with *norm FMR1* genotype. A numerical presentation of these data is presented in (a), In description of genotypes *norm* stands for normal, *het* for heterozygous and *hom* for homozygous. In description of sub-genotypes *high* stands for CGG_ n>34_ and *low* for CGG_ n<26._

Controls demonstrated a similar *FMR1* genotype and sub-genotype distribution to previously reported populations [4], [6] (Figure 1), with normal (*norm*), heterozygous (*het*) and homozygous (*hom*) genotypes of 58.0, 36.1 and 5.9 percent, respectively. The expected distribution was also observed with sub-genotypes, with *het-norm/high* at 15.6 and *het-norm/low* at 20.5 percent, each, and also follows that in general populations [4], [6].

Table 1 offers a detailed description of *BRCA1/2* mutations in the study group. *BRCA1/2* carriers presented with distinctively different *FMR1* genotype and sub-genotype distributions (Figure 1): An overwhelming majority of *BRCA1*/*2* patients exhibited the *het-norm/low FMR1* sub-genotype (74.0% *BRCA1* and 83.7% *BRCA2*, respectively). Combined, 78.8 percent of women who were *BRCA1* or *BRCA2* positive, thus, exhibited the *het-norm/low FMR1* sub-genotype. In further stark contrast to controls, none of the *BRCA1/2* carriers demonstrated the *het-norm/high FMR1* sub-genotype.

**Table 1:** Individual *BRCA1/2* mutations in study group.

Mutation type (n = 64)	Frequency count
1023delG	1
1135insA	1
1546dupCT	1
185delAG	1
1914del4	1
2041insA	2
2798delGAAA	1
3137delTTCA	3
3427delA	1
3473delGA	1
3600del11	2
3773delTT	1
4088delA	1
4143delT	1
4233insA	1
4512insT1428	1
4992del13	1
5343del5insG	1
5382insC	3
557ins25	1
5869delAAAT	2
5873C>A (S1882X)	2
5910C>G (Y1894X)	1
6174delT	1
6536C-A (S2103X)	1
6580delGT	1
6803del14	1
6869insC	1
703+3A-G (IVS5+3A>G)	1
7124insA	1
795delT	4
7994ins5	2
8034-2A-G (IVS16-2A>G)	1
8074delT	1
8230A-T (R2668X)	1
8592G-A (W2788X)	10
8715+1G>A (IVS19+1G>A)	1
886del GT	2
8983-1G>A (IVS21-1G>A)	5
9325insA	1
9610C>T(R3128X)	1
962del4	1
9900insA	1
C61G	2
del20–24	1
del5–14	1
dup11B	1
dup2	1
dup23	1
E755X	1
IVS16 -2A>G	1
IVS16+3G>C	1
IVS20 -1G>C	1
IVS2-1G>C	1
K1727X	1
L1086X	1
L639X	1
Q1395X	3
Q1424X	1
Q563X	5
R1203X	2
R1751X	2
R71M	1
W321X	1

None of the *BRCA1*/2 mutations demonstrated significant associations with *FMR1*. The single mutation noted in 10 patients was in 9 women associated with a *het-norm/low FMR1* sub-genotype.

*BRCA1/2*- positive patients also demonstrated almost no *norm* genotypes, by far the most prevalent genotype in controls (Figure 1) and in previously investigated populations [4], [6]: Only 10.0 and 2.0 percent of *BRCA1* and *BRCA2* patients, respectively (combined 6.1%), demonstrated a *norm FMR1* genotype.

The *hom* genotype was mildly overrepresented in *BRCA1* and *BRCA2* carriers (16.0% and 14.3%, respectively; combined, 15.2%). Numbers were, however, too small for meaningful assessments of individual *hom* sub-genotypes, and this group of patients was, therefore, collapsed. Controls had not been investigated for *BRCA1/2*.

**Figure 2:**
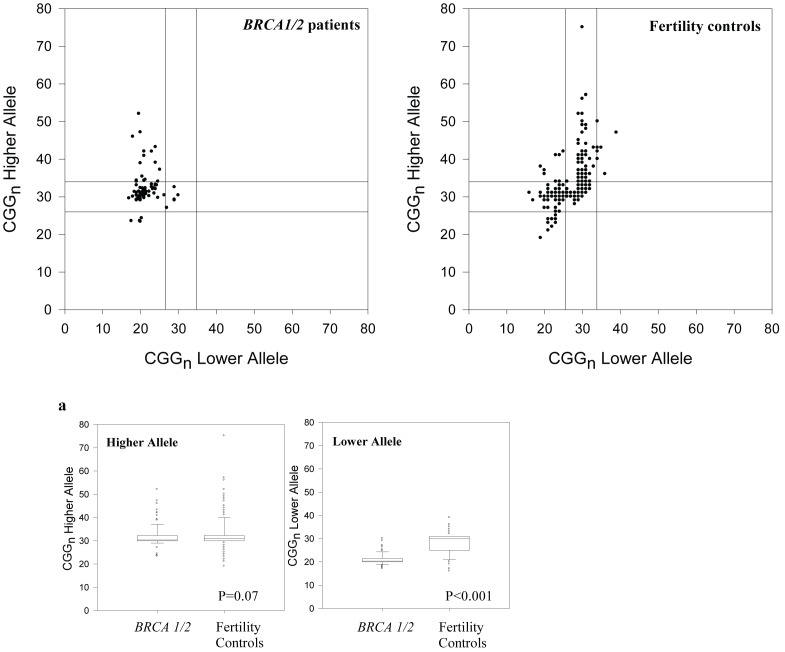
Distribution on both *FMR1* alleles, of CGG _n_ in *BRCA1/2* mutation carriers as well as U.S. controls in form of scattergrams. Horizontal and vertical parallel lines in scattergrams define the *norm* distribution area (CGG _n = 26–34_), with areas below and above representing *low* and *high*, sub-genotypes, respectively; a represents higher and lower count allele, respectively, for individual patients. Only the lower count allele varied significantly between the two groups (Mann-Whitney U test, P<0.001). Scattergrams, as well as a, demonstrate graphically the significant shift towards *low FMR1* sub-genotypes, especially on the lower count allele of *BRCA1/2* mutation carriers. In a - - - represents mean; ______ represents median.

In comparing distribution of *FMR1* genotypes and sub-genotypes between *BRCA1/2* patients and controls (with *hom* sub-genotypes collapsed), group membership was significantly related [x^2^ (6, N = 614)  = 158.71; P<0.0001). Non-parametric testing (Mann-Whitney U test)] confirmed statistically significant differences in median change for CGG_n_ on the low count allele of the *FMR1* gene between both patient groups; with follow up tests (Dunn’s Method) indicating significant differences between groups (Figure 2a & b).

For the lower CGG _n_ allele, in most cases representative of a *low FMR1* genotype/sub-genotype, values amongst the two groups were also significantly different [Mann-Whiney U = (Mean Rank 83.37_low,_ 296.44_ high;_ Z = −13.10; P = 0.001)]. The higher count CGG_ n_ allele, mostly representing *high FMR1* genotypes/sub-genotypes, varied amongst the two groups as well (Mann-Whitney U = Mean Rank 231.18_low_, 260.75_high_; Z = −0.069; P = 0.07) but failed to reach statistical significance (Figure 2a). Figure 2 presents distributions of individual CGG _n_ in both study groups.

## Discussion

This study was initiated to determine whether prematurely diminished functional ovarian reserve in women with *BRCA1/2* mutations, was, as had been suggested, a newly discovered association with *BRCA1/2* [8] or due to overlapping associations with *FMR1* genotypes and sub-genotypes, previously demonstrated to affect ovarian reserve [3]–[6]. Here reported finding answered this question rather unequivocally by demonstrating that *BRCA1/2* mutations were, practically, almost exclusively only associated with the *het-norm/low FMR1* sub-genotype. Since this sub-genotype has been associated with prematurely diminished ovarian reserve and lower pregnancy chances with IVF in all races [4], [6], it appears likely that the reported association of *BRCA1* with premature diminished ovarian reserve [8] is actually *FMR1*-mediated.

The here observed distribution of *FMR1* genotypes and sub-genotypes in *BRCA1/2* carriers came, however, as a complete surprise since no other previously investigated patient population had demonstrated a *FMR1* genotype/sub-genotype distribution as here observed amongst *BRCA1/2* carriers.

The most likely explanation for complete absence of *het-norm/high*, minimal presence of *norm* genotypes and highly excessive presence of *het-norm/low FMR1 sub*-genotypes in *BRCA1*/*2*-positive women is principal embryo-lethality of *BRCA1/2* mutations. Only if a human embryo carries a *low* (CGG_ n<26_) sub-genotype allele is such an embryo able to overcome the *BRCA1/2*-associated embryo lethality. Such *low* sub-genotypes can be present in *het-norm/low*, *hom-low/low* and *hom-high/low FMR1* sub-genotypes, combined, representing approximately 25 percent of all women (Figure 1) [4], [6]. In other words, only approximately one in four human embryos with *BRCA1/2* mutations will survive–a previously unreported cause of human embryo mortality.

An alternative explanation for here reported findings would be that *BRCA1/2* mutations, somehow, are able to influence CGG triple nucleotide repeats (CGG_n_) on the *FMR1* gene. Such an explanation, however, appears unlikely.

*BRCA1/2* mutations have never before in humans been reported to be embryo-lethal. Some homozygous *BRCA1/2* mouse models, however, proved embryo-lethal, though with great variability in phenotypes and in rescue of embryonic lethality on a p53-null background [12]. *BRCA1/2* genetically interacts with the p53 pathway, at least partially explaining the so-called “*BRCA* paradox,” defined by *BRCA-*deficient tumor cells rapidly proliferating, while *BRCA*-deficient embryos suffer from proliferation defects [12] (for further detail see later). In animal experiments, p53-nullizygousity can rescue some *BRCA1* mouse mutants [13]–[15] but may only delay lethality [16], [17]. The possibility of *BRCA1/2* being embryo lethal in humans, therefore, appears realistic.

This then raises the next important question: how do *low FMR1* sub-genotypes (CGG _n<26_) rescue embryos from *BRCA1/2* lethality? The answer will require a better understanding of the *FMR1* gene. Ovarian function associations of *het* sub-genotypes have been reasonably well defined [3], [4], [6]. The much rarer, *hom* sub-genotypes are less well defined and, here, had to be collapsed into a single group with potentially functionally opposing sub-genotypes.

Evolutionary, the *norm* genotype of *FMR1*, with both alleles in normal range (CGG _n = 26–34_), appears to represent the original (“*ur”*-) *FMR1* gene. Whether one or both alleles mutated outside of normal range, then determined *het* and *hom* genotypes. Expansions beyond CGG _n>34_ generated *high* sub-genotypes, primarily known for neuro-psychiatric risks in association with traditional premutation and full mutation genotypes [1], [2], [18]. Contractions to CGG _n<26_ resulted in *low* sub-genotypes with, as here demonstrated, rescue ability from embryo lethality by *BRCA1*/*2* mutations but increased risk towards autoimmunity [4], [6].

It is remarkable that not a single *BRCA1/2* patient demonstrated in this study a *high* (CGG _n>34_) sub-genotype, strongly suggesting that *high FMR1* sub-genotypes do not protect from embryo lethality. This is, however, not the first observation where *low* and *high* sub-genotypes of the *FMR1* gene denote opposing effects: *het-norm/high* was shown to be protective against autoimmunity, while *het-norm/low* promoted significant autoimmune risk [4], [6]. Since higher prevalence of autoimmunity in women has remained unexplained [19], the *FMR1* gene may have here an additional role to play.

Similar observations were recently also made for the polymorphic CAG repeat unit, which encodes an uninterrupted polyglutamine (polyQ) tract in the N-terminal transactivation domain of the androgen receptor. This is another prominent gene, characterized by ability to expand or contract trinucleotide repeat sequences from a normal range of CAG _n = 6–39_ [20]. Like with the *FMR1* gene, initial investigations only considered the gene’s expansion risk to be clinically significant. A recent study, however, for the first time found shorter CAG repeats associated with cryptorchidism risks [20].

Located at the 5′ untranslated exon 1 on the X chromosome at Xq27.3, a region now considered associated with autoimmune risks [21], the *FMR1* gene appeared positioned at crossroads of autoimmunity and reproduction [4]. Based on here reported data the gene, now, however, appears located at triple crossroads of autoimmunity, cancer and reproduction. Here is why: Lifetime risk for breast cancer of 1 per 8.2 women (12.2% per woman) increases in presence of *BRCA1/2* mutations approximately five-fold to ca. 60 percent (22). *BRCA1/2* mutations, thus, account for 5–10 percent of all breast cancers (23). Lifetime ovarian cancer risk of ca. 1.4 percent in presence of *BRCA1/2* mutations increases 10.7- to 28.6-fold to a 15 to 40 percent range [22]. *BRCA1/2*, thus, accounts for 10 to 15 percent of all ovarian cancer risk [23], with other cancers also demonstrating increased prevalence in association with *BRCA1* (uterine cervix and corpus, pancreas and colon) [24], [25] and *BRCA2* (pancreas, stomach, gallbladder, bile ducts and malignant melanoma) [26].

Because of high costs, *BRCA1/2* mutation screening is currently restricted to families at excessive risk for breast and ovarian cancers. Here presented data, if confirmed, suggest a potentially lower cost screening option via assessment of the *FMR1* gene since *BRCA1/2* positive women can, almost exclusively, be expected amongst approximately 25 percent of females with a *low FMR1* sub-genotype. The remaining approximately 75 percent of women, in turn, could be considered at only minimal risk to be *BRCA1*/2 carriers.

The estimated population frequency for *BRCA1/2* mutations (0.024 to 0.04%) in recessive and polygenic models, respectively [27], is held responsible for 5 to10 percent of all breast cancers and 10 to 15 percent of all ovarian cancer risk [23]. Extrapolating, the *het-norm/low FMR1* sub-genotype, representing approximately 78.8 percent of *BRCA1/2* patients, spread over only ca. a quarter of all women, would reflect 3.95 to 7.9 percent of all breast and 7.9 to 11.9 percent of ovarian cancer risk, concentrated in only approximately a quarter of the female population.

*Hom-low FMR1* sub-genotypes, *hom-high low* and *hom-low/low*, in this study not separately assessed, likely, would add a few percentage points.

*FMR1* genotypes and sub-genotypes [6] and prevalence of *BRCA1/2* mutations [28]–[30] vary in different races/ethnicities. Interestingly, so do female cancers [31], [32], autoimmunity [33] and female infertility prevalence [4]. It is tempting to hypothesize that these observations may be associated.

Since *BRCA* in normal cells induces growth arrest, while promoting tumor formation in *BRCA* mutation carriers, Evers and Jonkers pointed at the likely presence of secondary suppressor mutations, which may overcome *BRCA*-associated arrests during *BRCA*-associated tumorigenesis in association with the so-called “*BRCA* paradox” [12]. With *FMR1* apparently at crossroads of reproduction, immunology and cancer, it is tempting to hypothesize about such, each other opposing, functions for the two *het* sub-genotypes of *FMR1*, *het-norm/high* and *het-norm/low*. Within such a context *low FMR1* alleles not only may overcome embryo lethality (i.e., growth arrest) in human embryos but may also have a similar function in the induction of *BRCA1/2*-associated malignancies by overcoming the natural growth arrest functions of *BRCA1/2* by inducing tumor growth. Appropriate studies in *BRCA1/2*-associated tumor models, therefore, would be of interest.

Confirming such a growth arrest-reversing function of *low FMR1* alleles would, of course, have major relevance for the current understanding of tumor induction and diagnostic tumor risk assessments. Most importantly, however, one would have to conclude that in so-called *BRCA1/2*-associated tumors (like in premature decreased ovarian reserve) *low FMR1* alleles, and not *BRCA* mutation, are the real culprits. Finally, confirmation of such an *FMR1* function would rekindle decades-old considerations about common biological processes in pregnancy and malignancy [34], [35].

Here investigated patients were European and American, and, therefore, may reflect genetic diversities. Furthermore, their retroactive evaluations may have resulted in selection biases. Assay performance in different laboratories may have resulted in divergent results between study groups. Similarities in *FMR1* genotype and sub-genotype distribution between Austrian and U.S. control groups (for detail see Materials and Methods), however, practically rule out significant statistical impacts from laboratory or patient variability.

Remarkable statistical clarity of here reported results, therefore, strongly supports reported assertions, suggesting major new biological and clinical importance for *FMR1* and *BRCA1/2* mutations, deserving of further exploration.

## References

[1] Willemsen R, Levenga J, Oostra BA (2011) CGG repeat in the *FMR1* gene: size matters. Clin Genet 80: 214–25. 21651511 10.1111/j.1399-0004.2011.01723.x PMC3151325

[2] Wittenberger MD, Hagerman RJ, Sherman SL, McConkie-Rosell A, Welt CK, et al (2007) The FMR1 premutation and reproduction. Fertil Steril 87: 456–465. 17074338 10.1016/j.fertnstert.2006.09.004

[3] Gleicher N, Weghofer A, Barad DH (2010) Ovarian reserve determinations suggest new function of *FMR1* (fragile X gene) in regulating ovarian ageing. Reprod Biomed Online 20: 768–75. 20378415 10.1016/j.rbmo.2010.02.020

[4] Gleicher N, Weghofer A, Lee IH, Barad DH (2010) *FMR1* genotype with autoimmunity-associated polycystic ovary-like phenotype and decreased pregnancy chance. PLoS ONE 5: e15303. 21179569 10.1371/journal.pone.0015303 PMC3002956

[5] Gleicher N, Weghofer A, Barad DH (2010) Effects of race/ethnicity on triple CGG counts in the FMR1 gene in infertile women and egg donors. Reprod Biomed Online 20: 485–491. 20149747 10.1016/j.rbmo.2009.12.017

[6] Gleicher N, Weghofer A, Lee IH, Barad DH (2011) Association of *FMR1* genotypes with in vitro fertilization (IVF) outcomes based on ethnicity/race. PLoS ONE 6: e18781. 21526209 10.1371/journal.pone.0018781 PMC3078144

[7] Chen LS, Tassone FT, Sahota P, Hagerman PJ (2003) The (CGG)n repeat element within the 5’ untranslated region of the FMR1 message provides both positive and negative cis effects on in vivo translation of a downstream reporter. Hum Molec Genet 12: 3067–74. 14519687 10.1093/hmg/ddg331

[8] Oktay K, Kim JY, Barad D, Babayev SN (2010) Association of *BRCA1* mutations with occult primary ovarian insufficiency: a possible explanation for the link between infertility and breast/ovarian cancer risks. J Clin Oncol 28: 240–4. 19996028 10.1200/JCO.2009.24.2057 PMC3040011

[9] Wagner T, Stoppa-Lyonnet D, Fleischmann E, Muhr D, Pages S, et al (1999) Denaturing high-performance liquid chromatography detects reliably BRCA1 and BRCA2 mutations. Genomics 62: 369–76. 10644434 10.1006/geno.1999.6026

[10] Sanger F, Nickelen S, Coulson R (1977) DNA sequencing with chain-terminating inhibitors. Proc Natl Acad Sci USA 74: 5463–7. 271968 10.1073/pnas.74.12.5463 PMC431765

[11] Petek E, Kroisel PM, Schuster M, Zierler H, Wagner K (1999) Mosaicism in a fragile X male including a de novo deletion in the *FMR1* gene. Am. J Med Genet 84: 229–32. 10331598

[12] Evers B, Jonkers J (2006) Mouse models of BRCA1 and BRCA2 deficiency: past lessons, current understanding and future prospects. Oncogene 25: 5885–97. 16998503 10.1038/sj.onc.1209871

[13] Crook T, Crossland S, Crompton MR, Osin P, Gusterson BA (1997) p53 mutations in BRCA1-associated familial breast cancer. Lancet 350: 638–9. 10.1016/S0140-6736(05)63327-2 9288052

[14] Xu CF, Chambers JA, Nicolai H, Brown MA, Hujeirat Y, et al (1997) Mutations and alternative splicing of the BRCA1 gene in UK breast/ovarian cancer families. Genes Chromosomes Cancer 18: 102–10. 9115959

[15] Cao L, Li W, Kim S, Brodie SG, Deng CX (1993) Senescence, aging, and malignant transformation mediated by p53 in mice lacking the Brca1 full-length isoform. Genes Dev 17: 201–13. 10.1101/gad.1050003 PMC195980 12533509

[16] Hakem R, de la Pompa JL, Elia A, Potter J, Mark TW (1997) Partial rescue of Brca1 (5–6) early embryonic lethality by p53 or p21 null mutation. Nat Genet 16: 298–302. 9207798 10.1038/ng0797-298

[17] Ludwig T, Chapman DL, Papaioannou VE, Efstratiadis A (1997) Targeted mutations of breast cancer susceptibility gene homologs in mice: lethal phenotypes of Brca1, Brca2, Brca1/Brca2, Brca1/p53, and Brca2/p53 nullizygous embryos. Genes Dev 11: 1226–41. 9171368 10.1101/gad.11.10.1226

[18] Krausz C (2012) An encore for the repeats: New insights into an old genetic variant. J Clin Endocrinol Metab 97: 764–767. 22392953 10.1210/jc.2012-1130

[19] Gleicher N, Barad DH (2007) Gender as risk factor for autoimmune diseases. J Autoimmun 28: 1–6. 17261360 10.1016/j.jaut.2006.12.004

[20] Davis-Dao C, Koh CJ, Hardy BE, Chang A, Kim SS, et al.. (2012) Shorter androgen receptor CAG repeat lengths associated with cryptorchidism risk among Hispanic white boys. J Clin Endocrinol Metab 97;E393–E399. 10.1210/jc.2011-2439 22188741

[21] Gleicher N, Weghofer A, Barad DH (2012) Cutting edge assessment of the impact of autoimmunity on female reproductive success. J Autoimmun 38: J74–J80. 21664106 10.1016/j.jaut.2011.05.016

[22] Altekruse SF, Kosary CL, Krapcho M, et al. SEER Cancer Statistics Review 1975–2007, Bethesda, MD, National Cancer Institute (Accessed at http://seer.cancer.gov/csr/1975_2007/.)

[23] Campeau PM, Foulkes WD, Tischkowitz MD (2008) Hereditary breast cancer: new genetic developments, new therapeutic avenues. Human Genetics 124: 31–42. 18575892 10.1007/s00439-008-0529-1

[24] Kadouri L, Hubert A, Rotenberg Y, Hamburger T, Sagi M, et al (2007) Cancer risks in carriers of the BRCA1/2 Ashkenazi founder mutations. J Med Genet 44: 467–71. 17307836 10.1136/jmg.2006.048173 PMC2598014

[25] Thompson D, Easton DF (2002) Cancer Incidence in BRCA1 mutation carriers. J Nat Cancer Inst 94: 1358–65. 12237281 10.1093/jnci/94.18.1358

[26] The Breast Cancer Linkage Consortium (1999) Cancer risks in BRCA2 mutation carriers. J Nat Cancer Inst 91: 1310–6. 10433620 10.1093/jnci/91.15.1310

[27] Antoniou AC, Pharoah PD, McMullan G, Day NE, Ponder BA, et al (2001) Evidence for further breast cancer susceptibility genes in addition to BRCA1 and BRCA2 in a population-based study. Genet Epidemiol 21: 1–18. 11443730 10.1002/gepi.1014

[28] John EM, Miron A, Gong G, Phipps AI, Felberg A, et al (2007) Prevalence of pathogenic BRCA1 mutation carriers in 5 US racial/ethnic groups. JAMA 298: 2869–76. 18159056 10.1001/jama.298.24.2869

[29] Vogel KJ, Atchley DP, Erlichman J, Broglio KR, Ready KJ, et al (2007) BRCA1 and BRCA2 genetic testing in Hispanic patients: mutation prevalence and evaluation of the BRCAPRO risk assessment model. J Clin Oncol 25: 4635–41. 17925560 10.1200/JCO.2006.10.4703

[30] Malone KE, Daling JR, Doody DR, Hsu L, Bernstin L, et al (2006) Prevalence and predictors of BRCA1 and BRCA2 mutations in a population-based study of breast cancer in white and black American women ages 35 to 64 years. Cancer Res 66: 8297–308. 16912212 10.1158/0008-5472.CAN-06-0503

[31] Ademuyiwa FO, Edge SB, Erwin DO, Orom H, Ambrosone CB, et al (2011) Breast cancer racial disparities: unanswered questions. Cancer Res 71: 640–4. 21135114 10.1158/0008-5472.CAN-10-3021

[32] Terplan M, Schluterman N, McNamara EJ, Tracy JK, Temkin SM (2011) Have racial disparities in ovarian cancer increased over time? An analysis of SEER data. Gynecol Oncol Nov 21 [Epub ahead of print]. 10.1016/j.ygyno.2011.11.025 22108636

[33] Ikegami H, Kawabata Y, Noso S, Fujisawa T, Ogihara T (2007) Genetics of type 1 diabetes in Asian and Caucasian populations. Diabetes Res Clin Pract 77 Suppl 1S116–21. 17452059 10.1016/j.diabres.2007.01.044

[34] Gleicher N, Siegel I, Francus K (1980) Common denominators of pregnancy and malignancy. Mt Sinai J Med 47: 511–520. 7003362

[35] Gleicher N, Siegel I (1981) Common denominators of pregnancy and malignancy. Prog Clin Biol Res 70: 339–353. 7031695

